# *Cucurbits* Plants: A Key Emphasis to Its Pharmacological Potential

**DOI:** 10.3390/molecules24101854

**Published:** 2019-05-14

**Authors:** Bahare Salehi, Esra Capanoglu, Nabil Adrar, Gizem Catalkaya, Shabnum Shaheen, Mehwish Jaffer, Lalit Giri, Renu Suyal, Arun K Jugran, Daniela Calina, Anca Oana Docea, Senem Kamiloglu, Dorota Kregiel, Hubert Antolak, Ewelina Pawlikowska, Surjit Sen, Krishnendu Acharya, Zeliha Selamoglu, Javad Sharifi-Rad, Miquel Martorell, Célia F. Rodrigues, Farukh Sharopov, Natália Martins, Raffaele Capasso

**Affiliations:** 1 Student Research Committee, School of Medicine, Bam University of Medical Sciences, Bam 44340847, Iran; bahar.salehi007@gmail.com; 2 Faculty of Chemical & Metallurgical Engineering, Food Engineering Department, Istanbul Technical University, 34469 Maslak, Turkey; capanogl@itu.edu.tr (E.C.); catalkaya.gizem@gmail.com (G.C.); 3 Laboratoire de Biotechnologie Végétale et d’Ethnobotanique, Faculté des Sciences de la Nature et de la Vie, Université de Bejaia, Bejaia 06000, Algérie; n.adrar@hotmail.fr; 4 Department of Plant Sciences, LCWU, Lahore 54000, Pakistan; shabnum_shaheen78@hotmail.com (S.S.); meh.jaffer@gmail.com (M.J.); 5 G.B. Pant National Institute of Himalayan Environment & Sustainable Development Kosi-Katarmal, Almora 263 643, India; lalitorchid@gmail.com (L.G.); renusuyal04@gmail.com (R.S.); 6 G.B. Pant National Institute of Himalayan Environment & Sustainable Development Garhwal Regional Centre, Srinagar 246174, India; arunjugran@gbpihed.nic.in; 7 Department of Clinical Pharmacy, University of Medicine and Pharmacy of Craiova, 200349 Craiova, Romania; calinadaniela@gmail.com; 8 Department of Toxicology, University of Medicine and Pharmacy of Craiova, 200349 Craiova, Romania; daoana00@gmail.com; 9 Mevsim Gida Sanayi ve Soguk Depo Ticaret A.S. (MVSM Foods), Turankoy, Kestel, 16540 Bursa, Turkey; senemkamiloglu87@gmail.com; 10 Institute of Fermentation Technology and Microbiology, Lodz University of Technology, Wolczanska 171/173, 90-924 Lodz, Poland; dorota.kregiel@p.lodz.pl (D.K.); hubert.antolak@p.lodz.pl (H.A.); ewelina.pawlikowska@edu.p.lodz.pl (E.P.); 11 Molecular and Applied Mycology and Plant Pathology Laboratory, Department of Botany, University of Calcutta, Kolkata 700019, India; surjitsen09@gmail.com (S.S.); krish_paper@yahoo.com (K.A.); 12 Department of Botany, Fakir Chand College, Diamond Harbour, West Bengal 743331, India; 13 Department of Medical Biology, Faculty of Medicine, Nigde Ömer Halisdemir University, Campus, 51240 Nigde, Turkey; zselamoglu@ohu.edu.tr; 14 Zabol Medicinal Plants Research Center, Zabol University of Medical Sciences, Zabol 61615-585, Iran; 15 Department of Pharmacy, Faculty of Pharmacy, University of Concepcion, Concepcion 4070386, Chile; 16 LEPABE, Department of Chemical Engineering, Faculty of Engineering, University of Porto, Rua Dr. Roberto Frias, s/n, 4200-465 Porto, Portugal; c.fortunae@gmail.com; 17 Department of Pharmaceutical Technology, Avicenna Tajik State Medical University, Rudaki 139, Dushanbe 734003, Tajikistan; shfarukh@mail.ru; 18 Faculty of Medicine, University of Porto, Alameda Prof. Hernâni Monteiro, 4200-319 Porto, Portugal; 19 Institute for Research and Innovation in Health (i3S), University of Porto, 4200-135 Porto, Portugal; 20 Department of Agricultural Sciences, University of Naples Federico II, 80055 Portici, Italy

**Keywords:** cucurbits, pumpkin, squash, antimicrobial, antioxidant, anticancer, traditional medicine

## Abstract

*Cucurbita* genus has received a renowned interest in the last years. This plant species, native to the Americas, has served worldwide folk medicine for treating gastrointestinal diseases and intestinal parasites, among other clinical conditions. These pharmacological effects have been increasingly correlated with their nutritional and phytochemical composition. Among those chemical constituents, carotenoids, tocopherols, phenols, terpenoids, saponins, sterols, fatty acids, and functional carbohydrates and polysaccharides are those occurring in higher abundance. However, more recently, a huge interest in a class of triterpenoids, cucurbitacins, has been stated, given its renowned biological attributes. In this sense, the present review aims to provide a detailed overview to the folk medicinal uses of *Cucurbita* plants, and even an in-depth insight on the latest advances with regards to its antimicrobial, antioxidant and anticancer effects. A special emphasis was also given to its clinical effectiveness in humans, specifically in blood glucose levels control in diabetic patients and pharmacotherapeutic effects in low urinary tract diseases.

## 1. Introduction 

*Cucurbita* plants have been applied in different cultures as traditional medication. For instance, Native Americans have used pumpkins for the treatment of intestinal worms and urinary ailments, this therapeutic strategy being approved by American doctors in the early nineteenth century as an anthelmintic for worms annihilating [1]. Seeds are used as an anthelmintic, to treat issues of the urinary framework, high blood pressure, to prevent the development of kidney stones, to ease prostate disorders and even to improve the erysipelas skin contamination [2]. In southeastern Europe, *Cucurbita pepo* L. (pumpkin) seeds have been applied to heal irritable bladder and prostate enlargement. Specifically, in Germany, the use of pumpkin seeds was adopted for application by the authority for irritated bladder conditions and micturition problems of prostate enlargement, although the monograph written in 1985 noted a lack of pharmacological studies that could confirm its effective clinical effects. On the other hand, in the USA, the purchase of all such non-prescription medications for the therapy of prostate enlargement was banned in 1990. In traditional Chinese medicine, *Cucurbita moschata* Duchesne seeds were also applied for handling the parasitic diseases caused by worms, while Mexican herbalists have used *Cucurbita ficifolia* Bouché as a remedy for reducing blood sugar levels [3,4,5,6,7].

**Figure 1:**
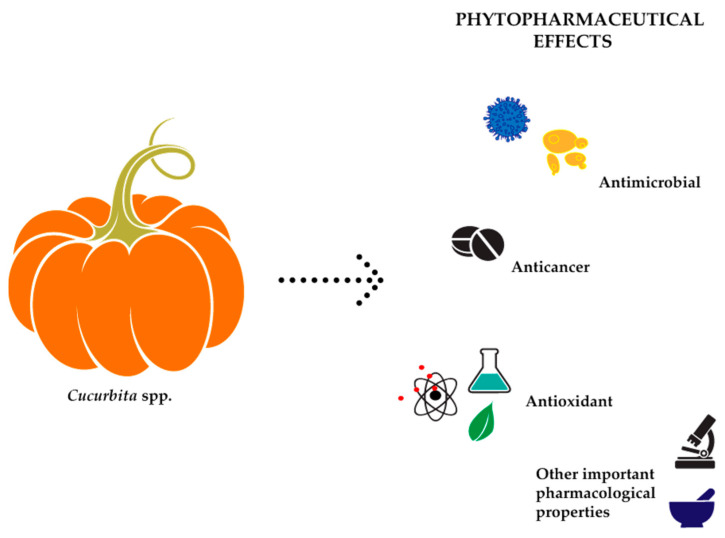
Most pronounced and investigated biological effects of *Cucurbita* spp.

Indeed, increasing evidence has shown that cucurbits’ medicinal properties depend upon the chemical compounds present, which produce a specific physiological effect in the human body [8,9,10]. Specifically, cucurbits fruits are found to be beneficial in blood cleansing, purification of toxic substances and good for digestion, besides giving the required energy to improve human health. These species possess a higher amount of proteins, phytosterols [11,12], unsaturated fatty acids [13,14], vitamins (like carotenoids, tocopherols) [15] and microelements (e.g., zinc) [16]. Fruits, seeds and leaves from various *Cucurbita* members (pumpkin, watermelon, melon, cucumber squash, gourds, etc.) possess different pharmacological effects [17,18], such as antidiabetic [19,20,21], antiulcer, analgesic, nephroprotective [22] and anticancer activities [18]. In this sense, this review provides a detailed overview to the folk medicinal uses of *Cucurbita* plants, an in-depth insight on the latest advances regarding its antimicrobial, antioxidant and anticancer effects, and lastly, a special emphasis to its clinical effectiveness in humans, specifically in blood glucose levels control and low urinary tract diseases (Figure 1).

## 2. *Cucurbita* Plants: A Brief Overview to Its Ethnopharmacological Uses

Recent ethnopharmacological studies showed that *C. pepo* and *Cucurbita maxima* Duchesne are among the most commonly used *Cucurbita* plants for traditional medicinal treatments. As shown in Table 1, many different components of *Cucurbita* plants are applied in diverse regions of the globe for handling different diseases.

In particular, the positive health effects of *C. maxima* seeds are well-documented [23,25,26,27,28]. Raw *C. maxima* seeds are orally administered for the treatment of digestive disorders, such as intestinal worms [23,25], constipation [23] and vomiting blood and blood bile [26] by the local people in the Iberian Peninsula, Argentina and India, respectively. Also, sun-dried seeds of *C. maxima* are ingested in Mauritius for the treatment of renal failure [27], whereas raw seeds are consumed to treat prostatitis in the Agro Nocerino Sarnese in Campania, Southern Italy [28]. *C. maxima* seeds, fruits, flowers and leaves are also used as traditional medicine [24,26,27,29,30,31], where the treatment of urinary disorders, blood pressure regulation and prevention of constipation can be achieved with oral consumption of *C. maxima* fruits, and the wound healing with dermal application [26,27,30]. In Mkuranga district in Tanzania, *C. maxima* leaves are used for healing anemia [24], and in the Ashanti region in Ghana, this plant part is orally consumed for lung and head cancer treatment [31]. Furthermore, in Mauritius, *C. maxima* fruits are compressed externally on eyes against cataract [27], while in India the same petals are used to treat osteosarcoma [29]. Nonetheless, and to the authors knowledge, much is needed to support both the in vitro and in vivo biological effects of this plant, since most of the efforts has been made towards its agro-industrial applications.

With regards to *C. pepo* seeds, they are mainly regarded as agro-industrial wastes, while in some parts of the globe they are used raw, roasted or cooked, at a domestic scale [40]. Accordingly, in a study carried out in Ghimbi District in Southwest Ethiopia [32], it was reported that oral administration of cultivated seed of *C. pepo* is used as a gonorrhea therapy. Moreover, *C. pepo* seeds are also used as an herbal remedy by breast cancer patients in West Bank in Palestine [36]. In another study, conducted in Nkonkobe municipality in Eastern Cape, South Africa [35], it was indicated that arthritis and blood booster are treated with orally taken *C. pepo* leaves. *C. pepo* leaves are also used for the treatment of malaria and dandruff in the local government area in south-eastern Nigeria and Ghimbi District in Southwest Ethiopia, respectively [37,38]. In the latter study, it was also pointed out that the fruits of *C. pepo* are consumed to treat gastritis and stomachache [37]. Topical use of *C. pepo* fruit as an external antiseptic was reported in Ripollès district, the Pyrenees in Catalonia and Iberian Peninsula, whereas in the same location the flowers of this plant are used for antigenic, antidermatitic, antiecchymotic, antiophidian, antipyretic and anti-toxic purposes [34]. *C. pepo,* as the whole plant, is also applied in the folk medicine of Mesoamerica and Caribbean for the therapy of fitness due to its pancreatic lipase inhibition activity [33]. In addition to the above, the decoction prepared from the *Cucurbita galeottii* Cogn. seeds is used against mucous discharge in Mauritius [39].

## 3. *Cucurbita* Plants Phytochemical Composition 

Carotenoids are highly present in the fruit of these plants, namely α-carotene, β-carotene, ζ-carotene, neoxanthin, violaxanthin, lutein, zeaxanthin, taraxanthin, luteoxanthin, auroxanthine, neurosporene, flavoxanthin, 5,6,5′,6′-diepoxy-β-carotene, phytofluene, α-cryptoxanthin and β-cryptoxanthin [41]. Total carotenoid content varied between 234.21 μg/g to 404.98 μg/g in *C. moschata* fruit [42], and 171.9 μg/g to 461.9 μg/g in *C. pepo* fruit [43]. There are also several publications on the carotenoid content of a number of *Cucurbita* plants such as *C. moschata*, *C. pepo* [42] and *C. maxima* [44]. Edible *Cucurbita* seeds are also rich in vitamin E (49.49 μg/g to 92.59 µg/g), γ-tocopherol is more abundant than α-tocopherol and the fruit contains less [45].

The study of Yang et al. [46] showed no flavonoid content (below detection limit: 0.05 mg/100 g) in either the immature or the mature fruit of *C. maxima*. Only the shoots and buds showed positive results. Sreeramulu and Raghunath [47] reported that average total phenolic content of *C. maxima* was 46.43 mg gallic acid equivalent (GAE)/100 g. In another study, *C. maxima* was analyzed for its flavonoid content and kaempferol was found to be the only flavonoid in this species at a concentration of 371.0 mg/kg of dry weight [48].

*C. pepo* was found to be very weak in polyphenol content. Only 0.02 mg GAE/100 mg sample has been found in its fresh fruit by Mongkolsilp et al. [49]. However, Iswaldi et al. [50] have reported for the first time a list of 34 polyphenols including a variety of flavonoids in the fruit of *C. pepo*., in addition to other unknown polar compounds. Besides, the flowers of *C. pepo* may contain considerable amount of phenolic compounds. Andjelkovic et al. [51] studied the phenolic content of six pumpkin (*C. pepo*) seed oils and identified the following compounds: Tyrosol, vanillic acid, vanillin, ferulic acid and luteolin. Among them, tyrosol was the most abundant compound ranging from 1.6 mg/kg to 17.7 mg/kg.

Peričin et al. [52] studied the phenolic acid content of *C. pepo* seeds. *p*-Hydroxybenzoic acid was found to be the prevailing phenolic acid, with 34.72%, 67.38% and 51.80% of the total phenolic acid content in whole dehulled seed, kernels and hulls, respectively. Aside from p-hydroxybenzoic acid, the most dominant phenolic compounds can be listed in a decreasing order of quantity as follows: Caffeic, ferulic and vanillic acids in whole dehulled seeds. *Trans*-synapic and protocatechuic acids, and *p*-hydroxybenzaldehyde were the abundant phenolic acids presented in the kernels of hulled pumpkin variety; the hulls comprised p-hydroxybenzaldehyde, vanillic and protocatechuic acids with considerable amounts. Table 2 presents the main phenolic compounds found in the *Cucurbita* spp. and their structures.

## 4. Looking at Cucurbita Plants Biological Activity

### 4.1. Antimicrobial Activity of Cucurbita Plants 

#### 4.1.1. In Vitro Studies

Pumpkin extracts showed a positive activity towards bacterial and fungal infections. They were effective against gram-positive: *Staphylococcus aureus, Bacillus subtilis,* as well as gram-negative bacterium: *Escherichia coli, Proteus vulgaris, Pseudomonas aeruginosa, Salmonella* spp. or *Klebsiella* spp. Pumpkin extracts also showed antibacterial activity against water borne bacteria *Vibrio cholerae* as well as intestinal flagellated parasite *Giardia lamblia*, often isolated from surface water. Other studies documented that pumpkin extracts showed a wide range of antifungal activity against species from the *Fusarium, Trichoderma, Aspergillus, Verticillium, Phytophora, Botrytis, Candida* and *Saccharomyces* genera (Table 3). However, the mechanisms of antimicrobial activity of pumpkin extracts are still unknown, although it seems to exist a synergistic action between all extracted bioactive substances. It is well known that plant extracts exert biological effects more prominent than their isolated compounds. In fact, recent evidence has established that, in whole matrices, the major compounds interact with those in trace amounts to potentiate their own potential, to provide additional properties other than those often recommended and even to help counterbalance the side effects of these isolated compounds. In addition, and not the least important to emphasize, is that such minor compounds by strengthening the biological effects of a specific bioactive also reduces the dose required to achieve a similar effect.

#### 4.1.2. In Vivo Studies

Not only pumpkin extracts, but also proteins and peptides isolated from *Cucurbita* spp. were identified and characterized in terms of antimicrobial activity. Three pumpkin proteins inhibited the growth of fungi *Fusarium oxysporum, Verticillium dahliae* and *Saccharomyces cerevisiae* [68]. The antifungal peptide—cucurmoschin—isolated from black pumpkin seeds also demonstrated inhibitory activity against mold growth: *Botrytis cinerea*, *F. oxysporum* and *Mycosphaerella oxysporum* [69]. The ribosome-inactivating protein extracted from *C. moschata* showed an antimicrobial effect towards phytopathogenic fungi *Phytophora infestans* as well as against bacteria *Pseudomonas solanacearum* and *Erwinia amylovora* [70]. Additionally, PR-5 protein isolated from leaves of pumpkin, demonstrated synergism with combination of nikkomycin, a chitin synthase inhibitor, towards to *Candida albicans* [71]. Protein Pr-1 isolated from pumpkin rind inhibited the growth of plant pathogenic fungi, namely *B. cinerea*, *F. oxysporum*, *F. solani* and *Rhizoctonia solani*, as well as the opportunistic pathogenic yeast *C. albicans* [72]. These results demonstrate that the proteins from pumpkin may be of importance to clinical microbiology with a wide range of therapeutic applications (Table 4). As the most prominent ones, and given the current evidence, namely regarding its ability to trigger fungal membranes damages and to improve the plasma membranes permeability, they can be effectively used to combat fungal infections and even to use in combination with current antifungal agents, both to improve its effectiveness and even to reduce its side effects.

Pumpkin pulp, due to its antimicrobial properties, is widely used to relieve intestinal inflammation or stomach disorders [73] (Table 5). Pumpkin and its seeds, in the traditional world medicine, are often employed as an anti-helminthic remedy and for supportive therapy in functional diseases of the bladder as well as in the case of digestion problems. The usage of an extract of *C. pepo* cortex towards urinary tract infections may correspond to a new source of antibiotics against bacterial urinary tract infections [57]. Other studies represented the importance of oil from seeds of a pumpkin as a hopeful drug for treating wounds in vivo [74]. The researchers demonstrated a premium quality of pumpkin oil with a high quantity of polyunsaturated fatty acids, tocopherols that were able to perform efficient wound healing [74]. Morphometric evaluation and histological evidence in rats showed healed biopsies from pumpkin oil and a complete re-epithelialization with a recurrence of skin appendages and well re-growing collagen fibers out of cells inflammation.

Pumpkin-based foodstuff is well recognized as a source of anti-inflammatory remedies, which can be useful in arthritis treatment [75]. Pumpkin seed oil notably prevent adjuvant-induced arthritis in rats, similar to indomethacin, a well-known anti-inflammatory substance. Its clinical applicability as an antioxidant was also assessed on rheumatoid arthritis [76] and recently confirmed by Dixon [77].

### 4.2. Anticancer Activities of Cucurbita Plants

Cucurbitacins are a distinct class of triterpenoids characterized by a cucurbitane-based structure, which contributes to their diverse biological activities, particularly their anticancer potential. Cucurbitacins have been identified as major secondary metabolites within the Cucurbitaceae family. They possess a biogenetically derived 10α-cucurbit-5-ene [19(10→19β) abeo-10α-lanostane] skeleton, which is closely linked to their cytotoxic effects. Several studies have attributed both in vitro and in vivo cytotoxic effects to cucurbitacins [79,80]. Jayaprakasam et al. [81] demonstrated the anticancer properties of cucurbitacins B, D, E and I, isolated from *Cucurbita andreana* Naudin, against colon, breast, lung, and central nervous system cancer cell lines. Among these, cucurbitacin B has been extensively investigated, with multiple studies confirming its efficacy in various cancer models, including in vivo tumor xenografts [82,83,84,85]. The precise mechanisms underlying its anticancer activity remain debated. The suppression of the oncogene Signal Transducer and Activator of Transcription 3 (STAT3) appears to play a key role in tumor growth inhibition [86], although alternative mechanisms may also contribute. Cancer remains a leading cause of mortality, accounting for approximately 12% of global deaths. Current therapeutic options include chemotherapy, surgical interventions, and radiation therapy. However, chemotherapy is often limited by drug resistance, toxicity, side effects, and insufficient selectivity for tumor cells [87]. Consequently, there is significant interest in exploring plant-derived bioactive compounds as promising sources for novel anticancer agents.

#### In Vitro Anticancer/Antitumor Effects

To date, over forty cucurbitacins have been isolated from the Cucurbitaceae family and other related medicinal plants. The proapoptotic effects of cucurbitacins are attributed to their ability to modulate gene expression, transcriptional activity via nuclear signaling, and mitochondrial membrane potential. Additionally, cucurbitacins can either activate or inhibit key apoptotic regulators. They are potent inhibitors of the JAK/STAT signaling pathway and also impact alternative apoptotic pathways, including PARP cleavage, MAPK signaling, and caspase-3 activation. Cucurbitacins have been shown to downregulate JAK3 and pSTAT3 levels, as well as several STAT3-regulated proteins involved in cell cycle progression, such as Bcl-2, Mcl-1, cyclin D3, and Bcl-xL [88]. *C. pepo* alcohol extract demonstrated cytotoxic activity against HepG2 and CT26 cancer cell lines, with IC_50_ values of 132.6 µg/mL and 167.2 µg/mL, respectively. Similarly, the ethanol extract of *C. pepo* exhibited a dose-dependent inhibitory effect on HeLa cell proliferation [89]. Cucurbita glycosides A and B, isolated from C. pepo ethanol extract, exhibited cytotoxic activity in vitro against HeLa cells, with IC_50_ values of 17.2 µg/mL and 28.5 µg/mL, respectively [90]. Cucurbitacins B and E, isolated from *C. pepo* cv dayangua, demonstrated antiproliferative effects against MCF-7, HCT-116, SF-268, A549, and NCI-H460 cancer cell lines [81]. The antiproliferative effects of 23,24-dihydrocucurbitacin F on human prostate cancer (PCa) cells may be attributed to the induction of cofilin–actin rod formation, leading to actin aggregation, cytokinesis failure, and cell cycle arrest at the G2/M phase, followed by apoptosis [91]. Furthermore, 23,24-dihydrocucurbitacin F has been shown to inhibit Epstein–Barr virus activation, induced by the tumor-promoting agent 12-O-tetradecanoylphorbol-13-acetate (TPA), and exerts significant antitumor-promoting effects in murine skin cancer models [88]. 

Treatment with cucurbitacins B and E resulted in apoptosis and cell cycle arrest in MDA-MB-231 and MCF-7 breast cancer cell lines. Additionally, these compounds modulated the expression of proteins involved in cell cycle regulation in both estrogen-independent (MDA-MB-231) and estrogen-dependent (MCF-7) human breast cancer cell lines. Cucurbitacin B was found to inhibit tumor growth and exert cytotoxic effects on SKBR-3 and MCF-7 breast cancer cell lines, primarily through G2/M phase arrest and apoptosis. Cucurbitacin B treatment also downregulated the expression of Cyclin D1, c-Myc, and β-catenin and prevented the nuclear translocation of β-catenin and galectin-3. Western blot analysis revealed increased PARP cleavage, suggesting caspase activation, and a reduction in Wnt signaling-related proteins such as galectin-3, β-catenin, c-Myc, and cyclin D1, along with modifications in phosphorylated GSK-3β levels [92]. 

Cucurbitacin E disrupted the cytoskeletal architecture of actin and vimentin, thereby inhibiting prostate cancer cell proliferation. Additionally, cucurbitacins suppressed endothelial cell proliferation by disrupting F-actin and tubulin microfilaments. Moreover, they exhibited antiangiogenic and antimetastatic properties by reducing T-lymphocyte proliferation and cellular motility [93]. Current research suggests that secondary metabolites from *C. pepo* possess significant anticancer activity, highlighting their potential role in the development of novel chemotherapeutic agents for tumor prevention and treatment.

## 5. Clinical Effectiveness of *Cucurbita* Plants in Humans 

### 5.1. Control of Blood Glucose Level in Diabetic Patients

*Diabetes mellitus* is a chronic disease characterized by changes in saccharide, lipid and protein metabolism resulting from a deficiency in insulin secretion from the pancreas, insulin resistance or both. The main clinical symptom is represented by increased blood sugar levels (hyperglycemia) that uncontrolled lead in time to a wide spectrum of complications [94]. Natural therapeutic alternatives to allopathic treatment always attracted the researchers to the intention of finding new drugs with fewer side effects [95,96,97,98,99,100]. Thus, the hypoglycemic effect of *Cucurbita* species (Table 6) is known and used for long traditional medicine in many countries, like China, India, Iran and Mexico [101,102,103,104]. 

Mahmoodpoor et al. [106] in a recent study performed on patients with severe diabetes from the Intensive Care Unit showed the hypoglycemic effect of *C. maxima* pulp. The subjects received five grams of *C. maxima* powder per 12 h for three consecutive days. After the treatment, it was observed a decrease of serum glucose levels from 214.9 mg/dL to 214.9 mg/dL associated with a reduction of insulin doses from 48.05 IU to 39.5 IU [106]. *C. ficifolia* also showed a good hypoglycemic effect when the extract was administered in doses of 4 mL/kg to patients with type 2 diabetes and moderately elevated blood glucose level [105]. Five hours after administration, the mean of serum glucose level decreased from 217.2 mg/dL to 150.8 mg/dL [105].

The most important hypoglycemic active substances in pumpkin are non-pectines polysaccharides and pectines from pulp, proteins and oil obtained from seeds [107,108,109]. Alenazi et al. [118] reported a clinical case of a 12-year-old Asian diabetic patient that ate every day for four months 200 g of pumpkin. After two months of daily pumpkin consumption, a decrease of glycosylated hemoglobin (HbA1C) from 10.8% to 8.5% was observed [118]. The same positive hypoglycemic effect was also revealed in another study by Jain et al. [119]. Fourteen patients diagnosed with type 2 diabetes received *C. ficifolia* juice for 40 days, and glycosylated hemoglobin decreased with 22.5% [119]. Shi et al. [120] investigated the antidiabetic activity of pumpkin carbohydrate granules in patients with type 2 diabetes compared to a control placebo group. After one month of treatment, both blood and urine glucose levels were significantly decreased compared with the placebo control group [120]. The results of a randomized, placebo-controlled trial conducted showed that a rich diet in pumpkin (*C. maxima*) seeds significantly reduced postprandial blood glucose of adults with normal glycaemia [121]. This study included 25 normoglycemic adults who consumed daily 65 g of pumpkin seeds [121]. Possible mechanisms of antihyperglycemic action of *Cucurbita* species are not fully understood but several studies investigated this subject in the last decades. Zhang et al. [122] demonstrated that *C. moschata* heteropolysaccharides regenerate pancreatic islets by stimulating proliferation of pancreatic β-cells. Quanhong et al. [123] showed that polysaccharides bounded by protein (polysaccharide 41.21% and protein 10.13%) increase glucose tolerance level and reduce hyperglycemia. In the light of these results, supplements with natural extracts from *Cucurbita* plants can be considered as alternative hypoglycemic products and further multicenter randomized studies can confirm these results.

### 5.2. Pharmacotherapeutic Effects in Low Urinary Tract Diseases

Benign prostatic hyperplasia (BPH) represents an increase in the volume of the prostate under the influence of androgenic hormones, and 70% of aging men suffer from this condition. Since clinical evolution of urinary signs is slow, prevention of BPH is useful, phytotherapy being an alternative way [124]. For example, oil obtained from *C. pepo* seeds is traditionally used to treat urinary symptoms in BPH as the daily frequency of urination, nycturia, time of the bladder emptying and residual volume [110,111]. The main mechanism through which these effects are obtained is represented by the inhibition of 5-α-reductase. This enzyme is required to convert testosterone to dihydrotestosterone, which has a higher affinity than testosterone for androgen receptors. As a result, protein synthesis increases the volume of the prostate implicitly [113].

In a multicenter clinical trial, thousands of patients diagnosed with BPH were treated with capsules containing 500 mg of *C. pepo* seeds extracts. Their quality of life has been significantly improved by reducing the urinary symptoms of BPH [125].

Other modern studies have shown pharmacotherapeutic synergism in BPH when *C. Pepo* is administrated simultaneously with other plants. Thus, the combination with *Serenoa repens* (W. Bartram) Small significantly improved the urinary symptoms of BPH and decreased blood dihydrotestosterone levels [111]. Hong et al. [112] obtained similar results on urinary symptoms in Korean men with BPH treated with 320 mg of *C. pepo* plus 320 mg of *S. repens*. They also observed a decrease in prostatic antigen levels after the treatment, but without changes in prostate volume [112]. In a randomized Phase II clinical trial carried out by Coulson et al. [126] the efficacy of the ProstateEZE Max formulation obtained from a mixture of plants traditionally used in treating BPH was evaluated. ProstateEZE is a natural formulation containing *C. pepo*, *S. repens, Pygeum africanum* Hook.f., *E. parviflorum* Schreb. and lycopene. Fifty-seven male patients diagnosed with BPH were selected in the study. Thirty-two of them received a capsule of ProstateEZE Max daily for three consecutive months, and 25 patients were treated with a placebo. In patients treated with Prostate EZE, the clinical symptoms of BPH decreased by 35.9% compared with only 8.3% for the placebo. The frequency of nocturnal urination was reduced with 39.3% in subjects treated for three months with ProstateEZE compared to the placebo group [126].

Due to these beneficial therapeutic effects of *Cucurbita* plants in BPH, the European Medicines Agency approved the use of *C. pepo* for both BPH and other bladder disorders, such as urinary stress incontinence in women [127].

Urinary stress incontinence occurs when pelvic muscles that support the bladder and the sphincter muscle, which controls the urinary flow, are weakened. This disorder is associated with aging in women. The main symptom is urinary incontinence [115]. The seeds extract of *C. pepo* have a therapeutic effect in this condition through a double mechanism. Directly by relaxing the bladder muscles leading to a decrease in nycturia and indirectly through a hormonal mechanism by inhibiting 5-α reductase. This inhibition determines the anabolic effects that strengthen the bladder sphincter muscles [115,127]. The main chemical compounds in the pumpkin seeds that explain these effects are sterols (sitosterol, spinasterol) and fatty oil, which contain oleic, linoleic, palmitic acids and tocopherol) [114]. Gažová et al. [128] demonstrated these effects in a study of 86 women with urinary incontinence stress who were treated for twelve weeks with the preparation of a plant mix: *C. pepo, Equisetum arvense* L. and *Linum usitatissimum* L. Episodes of urinary incontinence during the day were reduced to 35% and nocturnal urinary frequency to 54% [128].

Overactive bladder syndrome (OAB) is characterized by the frequent urge to urinate during the day and night, followed by an involuntary loss of urine [116]. A human clinical trial conducted by Shim et al. (2014) investigated the efficacy and utility of Cucuflavone (tablets with a mixture of plant extracts 87.5% *C. pepo* seeds and 12.5% soy) in reducing OAB symptoms [116]. The active compounds of Cucuflavone are phenols (pyrogallol) and isoflavones (genistein, daidzin). One hundred and twenty patients were included in the study, divided into two groups: The Cucuflavone group and the placebo group. Patients from Cucuflavone group received two tablets twice a day (a total of 875 mg of *C. pepo* seed extract and 125 mg of soy extract daily) for twelve weeks. The final results of the investigation showed that urinary incontinence, the frequency of daily and nocturnal urination was statistically significantly reduced compared to the initial parameters [116]. In a recent investigation, Nishimura et al. obtained similar results. They confirmed the efficacy of *C. maxima* seeds oil on urinary disorders in OAB. Forty-five subjects with OAB were included and treated daily with 10 g of *C. maxima* seed oil for twelve weeks. At the end of the investigation, the frequency of average daily urination was reduced from 10.96 to 8.00 [117].

## 6. Conclusions and Future Perspectives

In short, the use of *Cucurbita* species and their active constituents in various clinical and pharmacological studies revealed the presence of multiple, effective and useful compounds, which provide the opportunity for further production of antidiabetic, analgesic, anti-inflammatory and cardioprotective drugs and foods. Indeed, the use of *Cucurbita* plants in the treatment of several diseases, including gastrointestinal disorders, intestinal parasites and hypertension, dates from a long time ago. The antimicrobial and antioxidant properties of these species have triggered a huge interest for multiple applications. First of all, free radicals are generated through various metabolic activities in the body, ultimately resulting in various deleterious diseases [99]. These diseases can be treated by supplementation of cucurbits as activities of some cucurbits are comparable with commercially available antibiotics. The present review markedly highlights that *Cucurbita* species have preventive and therapeutic abilities for treatment of different diseases. The presence of active phytochemicals in *Cucurbita* species further strengthens the opportunity for their application as an upcoming anticancer, antidiabetic, analgesic, anti-inflammatory and cardioprotective drugs, as well as foods. Finally, and not the least important, the application of Cucurbitaceae members in public health, as nutraceuticals is associated with great availability and a good safety profile. 

## Figures and Tables

**Table 1:** *Cucurbita* plants traditionally applied in the cures of different diseases in diverse regions of the world.

Scientific Name (Common Name)	Location	Local Name	Parts Used	Administration	Disease(s) Treatment	References
*Cucurbita maxima* Duchesne (Squash)	Basque Country, Iberian Peninsula	Kalabazea	Seeds	Oral	Digestive (Intestinal worms, Constipation)	[23]
	Mkuranga District, Tanzania	Maboga	Leaves	Oral	Anemia	[24]
	Polish people in Misiones, Argentina	Zapallo	Seeds	Oral	Intestinal parasites	[25]
	Nelliyampathy hills of Kerala, India	Parangi	Seeds	Oral	Vomiting blood, Blood bile	[26]
			Fruits	Oral	Urinal disorders	
	Mauritius	Giromon	Flowers	Dermal	Cataract	[27]
			Seeds	Oral	Renal failure	
			Fruits	Dermal	Wound	
	Agro Nocerino Sarnese, Campania, Southern Italy	Cocozza	Seeds	Oral	Prostatitis	[28]
	India	UNSP	Flowers	UNSP	Osteosarcoma	[29]
	Pakistani descent in Copenhagen, Denmark	Kadoo	Fruits	Oral	Blood pressure, constipation	[30]
	Ashanti region, Ghana	UNSP	Leaves	Oral	Cancer (lung, head)	[31]
*Cucurbita pepo* L. (Pumpkin)	Ghimbi District, Southwest Ethiopia	Buqqee	Seeds	Oral	Gonorrhea	[32]
	Mexico, Central America, Caribbean	Calabaza	Whole plant	Oral	Obesity	[33]
	Ripollès district, Pyrenees, Catalonia, Iberian Peninsula	Carbassa	Flowers	Dermal	Acne, Dermatitis, Ecchymosis, Fever, Toxicity, Wound	[34]
			Fruits	Dermal	Infection	
	Nkonkobe Municipality, Eastern Cape, South Africa	Imithwane	Leaves	Oral	Arthritis, Blood booster	[35]
	West Bank, Palestine	Kare’a	Seeds	Oral	Breast cancer	[36]
	Delanta, Northwestern Wello, Northern Ethiopia	UNSP	Fruits	Oral	Gastritis, Stomachache	[37]
			Leaves	Dermal	Dandruff	
	Local Government Area, south-eastern Nigeria	Okeugu	Leaves	Oral	Malaria	[38]
*Cucurbita galeottii* Cogn. (Pumpkin)	Mauritius	Giraumon	Seeds	Oral	Mucous discharge	[39]

UNSP: Unspecified.

**Table 2:** Main chemical structures of the phenolic compounds found in the *Cucurbita* spp.*.

Compound Name	Synonym(s)	Empirical Formula	Structure	References
**Protocatechuic acid**	3,4-Dihydroxybenzoic acid	C_7_H_6_O_4_	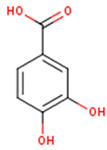	[52] http://phenol-explorer.eu/compounds/412
***p*-Hydroxybenzoic acid**	4-Hydroxybenzoic acid	C_7_H_6_O_3_	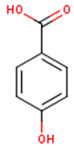	[52] http://phenol-explorer.eu/compounds/418
***p*-Hydroxybenzaldehyde**	4-Hydroxybenzaldehyde	C_7_H_6_O_2_	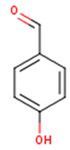	[52] http://phenol-explorer.eu/compounds/725
**Vanillic acid**	4-Hydroxy-3-methoxybenzoic acid; *p*-Vanillic acid	C_8_H_8_O_4_	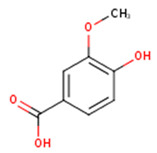	[52] http://phenol-explorer.eu/compounds/414
**Caffeic acid**	3,4-Dihydroxycinnamic acid	C_9_H_8_O_4_	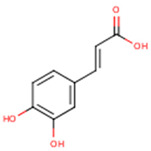	[52] http://phenol-explorer.eu/compounds/457
**Syringic acid**	3,5-Dimethoxy-4-hydroxybenzoic acid	C_9_H_10_O_5_	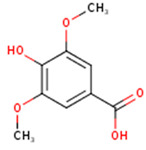	[52] http://phenol-explorer.eu/metabolites/420
***trans*-*p*-coumaric acid**	*trans*-4-Hydroxycinnamic acid	C_9_H_8_O_3_	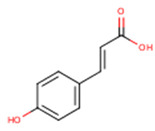	[52] http://phenol-explorer.eu/compounds/454
**Ferulic acid**	3-Methoxy-4-Hydroxycinnamic acid; 3-Methylcaffeic acid; Coniferic acid	C_10_H_10_O_4_	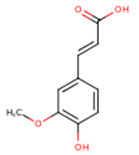	[52] http://phenol-explorer.eu/compounds/459
***trans*-sinapic acid**	*trans*-4-Hydroxy-3,5-dimethoxy-cinnamic acid; *trans*-Sinapinic acid	C_11_H_12_O_5_	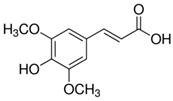	[52] http://phenol-explorer.eu/compounds/464
**Tyrosol**	*p*-HPEA; 4-(2-Hydroxyethyl)phenol; 2-(4-Hydroxyphenyl)ethanol; 2,4-Hydroxyphenyl-ethyl-alcohol; 4-Hydroxyphenylethanol	C_8_H_10_O_2_	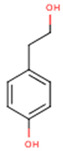	[52] http://phenol-explorer.eu/compounds/673
**Vanillin**	4-Hydroxy-3-methoxy-benzoic aldehyde; Methylprotocatechuic aldehyde; Vanillic aldehyde; *p*-Vanillin	C_8_H_8_O_3_	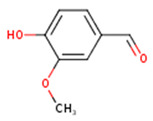	[52] http://phenol-explorer.eu/compounds/724
**Luteolin**	5,7,3′,4′-Tetrahydroxyflavone	C_15_H_10_O_6_	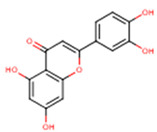	[52] http://phenol-explorer.eu/compounds/229
**Kaempferol**	3,5,7,4′-Tetrahydroxyflavone	C_15_H_10_O_6_	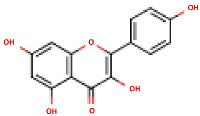	[52] http://phenol-explorer.eu/compounds/290

* The data were collected from the Phenol-Explorer database, which is an online comprehensive database on polyphenol contents in foods, http://phenol-explorer.eu/ (Accessed on 09.12.2018).

**Table 3:** Antimicrobial activity of *Cucurbita* spp. extracts evaluated in vitro.

*Cucurbita* spp./Plant Part	Extract	Microbial	References
*Cucurbita pepo* L. fruits	Water	*Escherichia coli*	[53]
*Cucurbita pepo* L. fruits	Methanol	*Bacillus cereus* *Bacillus subtilis* *Escherichia coli* *Enterobacter aerogenes* *Enterobacter agglomerans* *Salmonella enteritidis* *Salmonella choleraesuis* *Staphylococcus aureus* *Pseudomonas aeruginosa* *Enterobacter faecalis* *Klebsiella pneumoniae* *Bacillus sphericus* *Bacillus thruengenesis* *Cryptococcus meningitis* *Penicillium chrysogenum*	[54]
*Cucurbita pepo* L.	Phosphate buffered saline (PBS)	*Serratia marcescens* *Escherichia coli* *Streptococcus thermophilous* *Fusarium oxysporium* *Trichoderma reesei* *Aspergillus niger*	[55]
*Cucurbita pepo* L. fruits	Ethanol extract	*Heligmosoides bakeri* (worm)	[56]
*Cucurbita pepo* L. cortex	Water, methanol	*Staphylococcus aureus* *Escherichia coli* *Proteus mirabilis* *Klebsiella pneumoniae*	[57]
*Cucurbita pepo* L. seeds, backpeel	Methanol, ethanol	*Staphylococcus aureus* *Salmonella typhi*	[58]
*Cucurbita pepo* L. leaves	Ethanol	*Serratia* sp. *Escherichia coli* *Klebsiella pneumoniae* *Bacillus subtilis*	[59]
*Cucurbita pepo* L. leaves	Methanol	*Providencia stuartii* *Pseudomonas aeruginosa* *Klebsiella pneumoniae* *Escherichia coli* *Enterobacter aerogenes* *Enterobacter cloacae*	[60]
*Cucurbita pepo* L. leaves	Ethyl acetate, n-butanol, water	*Bacillus subtilis* *Pseudomonas aeruginosa* *Staphylococcus aureus*	[61]
*Cucurbita moschata* Duchesne seeds oil extract	Methanol	*Candida albicans* *Rhodotorula rubra* *Trichoderma viride* *Penicillium chrysogenum* *Rhizopus oligosporus*	[62]
*Cucurbita moschata* Duchesne crude protein from rinds, seeds and pulp	Acetone	*Aspergillus fumigatus* *Aspergillus parasiticus* *Aspergillus niger* *Staphylococcus aureus* *Bacillus subtilis* *Klebsiella pneumoniae* *Pseudomonas aeruginosa* *Escherichia coli*	[62]
*Cucurbita maxima* Duchesne fruit	Petroleum ether and methanol	*Giardia lamblia*	[63]
*Cucurbita maxima* Duchesne flowers	Alcohol	*Salmonella typhi,* *Escherichia coli* *Enterobacter faecalis,* *Bacillus cereus* *Curvularia lunata* *Candida albicans*	[64]
*Cucurbita maxima* Duchesne peels	Water	*Escherichia coli* *Pseudomonas* sp. *Vibrio cholerae*	[65]
*Cucurbita maxima* Duchesne seeds	Ethanol	*Entamoeba histolytica* *Staphylococcus aureus* *Bacillus subtilis* *Pseudomonas aeruginosa* *Escherichia coli* *Candida albicans* *Aspergillus niger*	[66]
*Cucurbita maxima* Duchesne seeds	Ethanol	*Staphylococcus aureus* *Bacillus subtilis* *Staphylococcus werneri* *Pseudomonas putida* *Pseudomonas aeruginosa* *Proteus mirabilis* *Escherichia coli* *Kleibsella pneumoniae*	[67]

**Table 4:** In vitro antimicrobial activity of *Cucurbita* spp. proteins.

*Cucurbita* spp. Proteins	Microbial	References
*Cucurbita maxima* Duchesne seeds proteins	*Fusarium oxysporum,* *Verticillium dahliae* *Saccharomyces cerevisiae*	[68]
*Cucurbita maxima* Duchesne seeds protein RIP	*Phytophora infestans,* *Erwinia amylovora,* *Pseudomonas solanacearum*	[70]
Pumpkin leaves protein PR-5	*Candida albicans*	[71]
Pumpkin rind protein Pr-1	*Botrytis cinerea,* *Fusarium oxysporum,* *Fusarium solani* *Rhizoctonia solani,* *Candida albicans*	[72]
Black pumpkin seeds protein cucurmoschin	*Botrytis cinerea,* *Fusarium oxysporum* *Mycosphaerella oxysporum*	[69]

**Table 5:** Antimicrobial property of *Cucurbita* spp. and its importance in vivo.

	Antimicrobial Property	References
*Cucurbita pepo* L. seeds	Wounds healing	[74]
Pumpkin seeds	Anthelmintic, treatment of bladder functional disorders	[78]
Pumpkin seed oil	Arthritis prevention	[75]
Pumpkin fruits	Control of gastrointestinal nematode infections	[56]
*Cucurbita pepo* L. cortex extract	Effective treatment of bacterial urinary tract infections	[57]

**Table 6:** Pharmacotherapeutic effects of *Cucurbita* plants in human clinical studies.

	Part of the Plant with Active Compounds	*Cucurbita* spp.	References
Hypoglycemic	Polysaccharides from pulp fruit	*Cucurbita maxima* Duchesne *Cucurbita ficifolia* Bouché	[105,106]
	Non-pectines polysaccharides and pectines from pulp; proteins and oil from seeds	*Cucurbita ficifolia* Bouché	[107,108,109]
Reduced clinical symptoms of benign prostatic hyperplasia	Δ5-Δ7-Δ8-Phytosterols, unsaturated fatty acids from seeds extracts, lignans	*Cucurbita pepo* L.	[110,111,112,113]
Positive effects in stress urinary incontinence in female	Oil, sterols from seeds	*Cucurbita pepo* L.	[114,115]
Improved urinary symptoms in human overactive bladder	Seeds oil (sterols) blended with soy germ extract (phenols, isoflavones)	*Cucurbita pepo* L.	[116]
		*Cucurbita maxima* Duchesne	[117]
