# Correction: Salehi et al. *Cucurbits* Plants: A Key Emphasis to Its Pharmacological Potential. *Molecules* 2019, *24*, 1854

**DOI:** 10.3390/molecules30122525

**Published:** 2025-06-10

**Authors:** Bahare Salehi, Esra Capanoglu, Nabil Adrar, Gizem Catalkaya, Shabnum Shaheen, Mehwish Jaffer, Lalit Giri, Renu Suyal, Arun K Jugran, Daniela Calina, Anca Oana Docea, Senem Kamiloglu, Dorota Kregiel, Hubert Antolak, Ewelina Pawlikowska, Surjit Sen, Krishnendu Acharya, Zeliha Selamoglu, Javad Sharifi-Rad, Miquel Martorell, Célia F. Rodrigues, Farukh Sharopov, Natália Martins, Raffaele Capasso

**Affiliations:** 1Student Research Committee, School of Medicine, Bam University of Medical Sciences, Bam 44340847, Iran; bahar.salehi007@gmail.com; 2Faculty of Chemical & Metallurgical Engineering, Food Engineering Department, Istanbul Technical University, 34469 Maslak, Turkey; capanogl@itu.edu.tr (E.C.); catalkaya.gizem@gmail.com (G.C.); 3Laboratoire de Biotechnologie Végétale et d’Ethnobotanique, Faculté des Sciences de la Nature et de la Vie, Université de Bejaia, Bejaia 06000, Algérie; n.adrar@hotmail.fr; 4Department of Plant Sciences, LCWU, Lahore 54000, Pakistan; shabnum_shaheen78@hotmail.com (S.S.); meh.jaffer@gmail.com (M.J.); 5G.B. Pant National Institute of Himalayan Environment & Sustainable Development Kosi-Katarmal, Almora 263 643, India; lalitorchid@gmail.com (L.G.); renusuyal04@gmail.com (R.S.); 6G.B. Pant National Institute of Himalayan Environment & Sustainable Development Garhwal Regional Centre, Srinagar 246174, India; arunjugran@gbpihed.nic.in; 7Department of Clinical Pharmacy, University of Medicine and Pharmacy of Craiova, 200349 Craiova, Romania; calinadaniela@gmail.com; 8Department of Toxicology, University of Medicine and Pharmacy of Craiova, 200349 Craiova, Romania; daoana00@gmail.com; 9Mevsim Gida Sanayi ve Soguk Depo Ticaret A.S. (MVSM Foods), Turankoy, Kestel, 16540 Bursa, Turkey; senemkamiloglu87@gmail.com; 10Institute of Fermentation Technology and Microbiology, Lodz University of Technology, Wolczanska 171/173, 90-924 Lodz, Poland; dorota.kregiel@p.lodz.pl (D.K.); hubert.antolak@p.lodz.pl (H.A.); ewelina.pawlikowska@edu.p.lodz.pl (E.P.); 11Molecular and Applied Mycology and Plant Pathology Laboratory, Department of Botany, University of Calcutta, Kolkata 700019, India; surjitsen09@gmail.com (S.S.); krish_paper@yahoo.com (K.A.); 12Department of Botany, Fakir Chand College, Diamond Harbour, West Bengal 743331, India; 13Department of Medical Biology, Faculty of Medicine, Nigde Ömer Halisdemir University, Campus, 51240 Nigde, Turkey; zselamoglu@ohu.edu.tr; 14Zabol Medicinal Plants Research Center, Zabol University of Medical Sciences, Zabol 61615-585, Iran; 15Department of Pharmacy, Faculty of Pharmacy, University of Concepcion, Concepcion 4070386, Chile; 16LEPABE, Department of Chemical Engineering, Faculty of Engineering, University of Porto, Rua Dr. Roberto Frias, s/n, 4200-465 Porto, Portugal; c.fortunae@gmail.com; 17Department of Pharmaceutical Technology, Avicenna Tajik State Medical University, Rudaki 139, Dushanbe 734003, Tajikistan; shfarukh@mail.ru; 18Faculty of Medicine, University of Porto, Alameda Prof. Hernâni Monteiro, 4200-319 Porto, Portugal; 19Institute for Research and Innovation in Health (i3S), University of Porto, 4200-135 Porto, Portugal; 20Department of Agricultural Sciences, University of Naples Federico II, 80055 Portici, Italy

## Text Correction

There was an error in the original publication [1]. Section 4.2. Anticancer Activities of Cucurbita Plants, contained uncommon terminology and linguistic inconsistencies that affected clarity and readability. This section has been thoroughly revised to improve clarity, accuracy, and adherence to standard scientific language while maintaining the original meaning. This revision was carried out with the assistance of a native English-speaking medical expert to ensure precise and appropriate terminology.

A correction has been made to Section 4.2. Anticancer Activities of Cucurbita Plants. The corrected text is as follows:


*4.2. Anticancer Activities of Cucurbita Plants*


Cucurbitacins are a distinct class of triterpenoids characterized by a cucurbitane-based structure, which contributes to their diverse biological activities, particularly their anticancer potential. Cucurbitacins have been identified as major secondary metabolites within the Cucurbitaceae family. They possess a biogenetically derived 10α-cucurbit-5-ene [19(10→19β) abeo-10α-lanostane] skeleton, which is closely linked to their cytotoxic effects. Several studies have attributed both in vitro and in vivo cytotoxic effects to cucurbitacins [79,80]. Jayaprakasam et al. [81] demonstrated the anticancer properties of cucurbitacins B, D, E and I, isolated from *Cucurbita andreana* Naudin, against colon, breast, lung, and central nervous system cancer cell lines. Among these, cucurbitacin B has been extensively investigated, with multiple studies confirming its efficacy in various cancer models, including in vivo tumor xenografts [82–85]. The precise mechanisms underlying its anticancer activity remain debated. The suppression of the oncogene Signal Transducer and Activator of Transcription 3 (STAT3) appears to play a key role in tumor growth inhibition [86], although alternative mechanisms may also contribute. Cancer remains a leading cause of mortality, accounting for approximately 12% of global deaths. Current therapeutic options include chemotherapy, surgical interventions, and radiation therapy. However, chemotherapy is often limited by drug resistance, toxicity, side effects, and insufficient selectivity for tumor cells [87]. Consequently, there is significant interest in exploring plant-derived bioactive compounds as promising sources for novel anticancer agents.

In Vitro Anticancer/Antitumor Effects

To date, over forty cucurbitacins have been isolated from the Cucurbitaceae family and other related medicinal plants. The proapoptotic effects of cucurbitacins are attributed to their ability to modulate gene expression, transcriptional activity via nuclear signaling, and mitochondrial membrane potential. Additionally, cucurbitacins can either activate or inhibit key apoptotic regulators. They are potent inhibitors of the JAK/STAT signaling pathway and also impact alternative apoptotic pathways, including PARP cleavage, MAPK signaling, and caspase-3 activation. Cucurbitacins have been shown to downregulate JAK3 and pSTAT3 levels, as well as several STAT3-regulated proteins involved in cell cycle progression, such as Bcl-2, Mcl-1, cyclin D3, and Bcl-xL [88]. *C. pepo* alcohol extract demonstrated cytotoxic activity against HepG2 and CT26 cancer cell lines, with IC_50_ values of 132.6 µg/mL and 167.2 µg/mL, respectively. Similarly, the ethanol extract of *C. pepo* exhibited a dose-dependent inhibitory effect on HeLa cell proliferation [89]. Cucurbita glycosides A and B, isolated from C. pepo ethanol extract, exhibited cytotoxic activity in vitro against HeLa cells, with IC_50_ values of 17.2 µg/mL and 28.5 µg/mL, respectively [90]. Cucurbitacins B and E, isolated from *C. pepo* cv dayangua, demonstrated antiproliferative effects against MCF-7, HCT-116, SF-268, A549, and NCI-H460 cancer cell lines [81]. The antiproliferative effects of 23,24-dihydrocucurbitacin F on human prostate cancer (PCa) cells may be attributed to the induction of cofilin–actin rod formation, leading to actin aggregation, cytokinesis failure, and cell cycle arrest at the G2/M phase, followed by apoptosis [91]. Furthermore, 23,24-dihydrocucurbitacin F has been shown to inhibit Epstein–Barr virus activation, induced by the tumor-promoting agent 12-O-tetradecanoylphorbol-13-acetate (TPA), and exerts significant antitumor-promoting effects in murine skin cancer models [88].

Treatment with cucurbitacins B and E resulted in apoptosis and cell cycle arrest in MDA-MB-231 and MCF-7 breast cancer cell lines. Additionally, these compounds modulated the expression of proteins involved in cell cycle regulation in both estrogen-independent (MDA-MB-231) and estrogen-dependent (MCF-7) human breast cancer cell lines. Cucurbitacin B was found to inhibit tumor growth and exert cytotoxic effects on SKBR-3 and MCF-7 breast cancer cell lines, primarily through G2/M phase arrest and apoptosis. Cucurbitacin B treatment also downregulated the expression of Cyclin D1, c-Myc, and β-catenin and prevented the nuclear translocation of β-catenin and galectin-3. Western blot analysis revealed increased PARP cleavage, suggesting caspase activation, and a reduction in Wnt signaling-related proteins such as galectin-3, β-catenin, c-Myc, and cyclin D1, along with modifications in phosphorylated GSK-3β levels [92].

Cucurbitacin E disrupted the cytoskeletal architecture of actin and vimentin, thereby inhibiting prostate cancer cell proliferation. Additionally, cucurbitacins suppressed endothelial cell proliferation by disrupting F-actin and tubulin microfilaments. Moreover, they exhibited antiangiogenic and antimetastatic properties by reducing T-lymphocyte proliferation and cellular motility [93]. Current research suggests that secondary metabolites from *C. pepo* possess significant anticancer activity, highlighting their potential role in the development of novel chemotherapeutic agents for tumor prevention and treatment.

## References

In the original publication, Reference [87] was found to be inappropriate for its context, as it primarily discussed food science rather than drug nanocarriers for chemotherapy. A correction has been made to Reference [87].

Old Citation:

Carvalho, L.J.; Smiderle, L.A.S.; Carvalho, J.L.V.; Cardoso, F.S.N.; Koblitz, M.G.B. Assessment of carotenoids in pumpkins after different home cooking conditions. *Food Sci. Technol.* **2014**, *34*, 365–370.

New Citation:

Pérez-Herrero, E.; Fernández-Medarde, A. Advanced targeted therapies in cancer: Drug nanocarriers, the future of chemotherapy. *Eur. J. Pharm. Biopharm.*
**2015**, *93*, 52–79. https://doi.org/10.1016/j.ejpb.2015.03.018.

The authors state that the scientific conclusions are unaffected. These correction were approved by the Academic Editor. The original publication has also been updated.
